# A Unique mRNA Vaccine Elicits Protective Efficacy against the SARS-CoV-2 Omicron Variant and SARS-CoV

**DOI:** 10.3390/vaccines12060605

**Published:** 2024-06-01

**Authors:** Xiaoqing Guan, Abhishek K. Verma, Gang Wang, Abhijeet Roy, Stanley Perlman, Lanying Du

**Affiliations:** 1 Institute for Biomedical Sciences, Georgia State University, Atlanta, GA 30303, USA; 2 Department of Microbiology and Immunology, University of Iowa, Iowa City, IA 52242, USA; 3 Department of Pediatrics, University of Iowa, Iowa City, IA 52242, USA

**Keywords:** coronavirus, COVID-19, SARS-CoV-2, SARS-CoV, spike protein, receptor-binding domain, unique mRNA vaccine

## Abstract

The highly pathogenic coronaviruses SARS-CoV-2 and SARS-CoV have led to the COVID-19 pandemic and SARS outbreak, respectively. The receptor-binding domain (RBD) of the spike (S) protein of SARS-CoV-2, particularly the Omicron variant, has frequent mutations, resulting in the reduced efficiency of current COVID-19 vaccines against new variants. Here, we designed two lipid nanoparticle-encapsulated mRNA vaccines by deleting the mutant RBD of the SARS-CoV-2 Omicron variant (SARS2-S (RBD-del)) or by replacing this mutant RBD with the conserved and potent RBD of SARS-CoV (SARS2-S (SARS-RBD)). Both mRNA vaccines were stable at various temperatures for different time periods. Unlike SARS2-S (RBD-del) mRNA, SARS2-S (SARS-RBD) mRNA elicited effective T-cell responses and potent antibodies specific to both SARS-CoV-2 S and SARS-CoV RBD proteins. It induced strong neutralizing antibodies against pseudotyped SARS-CoV-2 and SARS-CoV infections and protected immunized mice from the challenge of the SARS-CoV-2 Omicron variant and SARS-CoV by significantly reducing the viral titers in the lungs after Omicron challenge and by completely preventing SARS-CoV-induced weight loss and death. SARS2-S (SARS-RBD)-immunized serum antibodies protected naïve mice from the SARS-CoV challenge, with its protective efficacy positively correlating with the neutralizing antibody titers. These findings indicate that this mRNA vaccine has the potential for development as an effective vaccine against current and future SARS-CoV-2 variants and SARS-CoV.

## 1. Introduction

Severe acute respiratory syndrome-coronavirus (SARS-CoV) and SARS-CoV-2, two highly pathogenic coronaviruses (CoVs), have led to significant threats to global public health. SARS-CoV was initially identified in 2002, inducing a global outbreak with a mortality rate of 10%; whereas, SARS-CoV-2 was first identified in 2019 and caused the pandemic of COVID-19 (Coronavirus Disease 2019) [1,2,3,4,5]. SARS-CoV-2 has high transmissibility and presents frequent mutations, giving rise to several mutant variants, including the currently prevalent Omicron variant and its subvariants [6,7,8,9]. Many previously developed COVID-19 vaccines have diminished their neutralizing activity against these new variants [10,11,12]. A number of SARS-like CoVs use the receptor of SARS-CoV-2 and SARS-CoV for viral entry [1,13]. Thus, universal vaccines with broad-spectrum efficiency are urgently needed to prevent infections of not only SARS-CoV-2 and its variants but also SARS-CoV or SARS-like CoVs with pandemic potential.

SARS-CoV-2 and SARS-CoV are categorized in the subfamily *Coronavirinae*, the family of *Coronaviridae*, and the order of *Nidovirales* [14]. The surface spike (S) protein of SARS-CoV-2 and SARS-CoV is the most important structural protein causing viral infection and pathogenesis. The S protein binds to angiotensin-converting enzyme 2 (ACE2), the receptor of SARS-CoV-2 and SARS-CoV, through the receptor-binding domain (RBD) of the S1 subunit. Therefore, both the S protein and the RBD fragment of these CoVs are important vaccine targets [13,15,16,17]. The RBD of SARS-CoV is relatively conserved among different strains, so vaccines based on the SARS-CoV RBD may elicit broadly neutralizing antibodies against multiple SARS-CoV strains or SARS-related CoVs from other species [16,18]. Nevertheless, the RBD of SARS-CoV-2, particularly Omicron variants, mutates frequently. To date, 20 or more mutations have been identified in the RBD region of the SARS-CoV-2 Omicron variant and its subvariants, significantly affecting the ability of vaccines covering the mutant RBD region or antibodies targeting the mutant RBD epitopes to prevent various SARS-CoV-2 infections [6,11,19,20,21,22,23,24]. By contrast, the non-RBD region of the S protein of SARS-CoV has high sequence homology among different SARS-CoV-2 strains, including various Omicron subvariants. Therefore, it is hypothesized that universal vaccines with broad-spectrum efficacy can be developed by targeting the conserved S protein of SARS-CoV-2 with the insertion of a heterologous immunogenic RBD of SARS-CoV.

Generally, mRNA vaccines delivered via lipid nanoparticles (LNPs) have unique properties with effective stability, fast rate, low cost, and large-scale capability [25,26]. A number of mRNA vaccines that target SARS-CoV-2 RBD have been developed, which include those that target the ancestral strain and some variants, such as Omicron BA.4/5 subvariants, and their neutralizing activity and/or protective efficacy against different strains vary, depending on the SARS-CoV-2 strains or animal models tested [27,28,29]. Although SARS-CoV-2 RBD-mRNA-induced antibodies may cross-neutralize SARS-CoV infection, the neutralizing activity is relatively low [30].

This study reported the design of two LNP-encapsulated mRNA vaccines by (1) deleting the mutant RBD region of the S protein of a SARS-CoV Omicron variant (SARS2-S (RBD-del)) or (2) by replacing its mutant RBD region with the RBD of SARS-CoV (SARS2-S (SARS-RBD)). Their stability, immunogenicity, and ability to protect against infection with the SARS-CoV-2 Omicron variant and SARS-CoV were compared. In addition, immune sera from vaccinated mice were passively transferred to naïve mice, with the latter mice subsequently challenged with SARS-CoV. These results showed that SARS2-S (SARS-RBD) has the potential for development as an effective mRNA vaccine against both SARS-CoV-2 and SARS-CoV infections.

## 2. Materials and Methods

### 2.1. Design and Construct of mRNA Vaccines

The mRNAs used in this study were designed and constructed as described below [30,31]. Specifically, the recombinant SARS2-S (RBD-del) DNA includes the S gene (removing its RBD) of the Omicron variant (GISAID accession number EPI_ISL_6795835) of SARS-CoV-2, and contains an N-terminal signal peptide, tissue plasminogen activator (tPA), and C-terminal foldon trimeric sequence and His_6_ tag. The codon-optimized S gene (synthesized by GenScript, Piscataway, NJ, USA), which consists of a HexaPro sequence (e.g., mutation of the furin cleavage site and six substitutions of proline residue) of the above SARS-CoV-2 Omicron variant, was enzyme digested and inserted into a pCAGGS expression vector. The recombinant SARS2-S (SARS-RBD) DNA was constructed by inserting the sequence of a codon-optimized RBD gene (synthesized by GenScript) of SARS-CoV (Tor2 strain; GenBank accession number AY274119) into the construct described above. The positive recombinant plasmids were confirmed for correct sequences by Sanger DNA sequencing (AZENTA, Burlington, MA, USA) and no unexpected mutations were identified after sequencing.

### 2.2. Synthesis of mRNA Vaccines

The mRNA vaccines were synthesized as described below [30,32]. Specifically, after linearizing each of the recombinant plasmids, the related mRNAs were synthesized according to the instruction of the MEGAscript T7 Transcription Kit (Thermo Fisher Scientific, Waltham, MA, USA). Pseudo-UTP (Ψ) (APExBIO, Houston, TX, USA) was added together with CTP, ATP, and GTP nucleosides to form nucleoside-modified mRNAs to increase mRNA’s stability. The synthesized mRNAs were subjected to the RNA Cleanup Kit (New England Biolabs, Ipswich, MA, USA) and then capped and tailed using the Cap 1 Capping System Kit and Poly(A) Polymerase Tailing Kit, respectively (CELLSCRIPT, Madison, WI, USA).

### 2.3. mRNA Formulation and Characterization

To further increase mRNA’s stability, the mRNAs synthesized above were formulated with LNPs and delivered in the form of mRNA-LNPs [12,30,31]. Specifically, the mRNAs synthesized above were incubated with PNI Formulation Buffer (NWW0043, Precision Nanosystems, Vancouver, BC, Canada), followed by encapsulation with lipids, GenVoy-ILM (NWW0042, Precision Nanosystems) at a ratio of 3:1, using NanoAssemblr Ignite Instrument according to the manufacturer’s protocols (Precision Nanosystems). The encapsulated mRNA-LNPs were diluted with sterile Ca^2+^ and Mg^2+^-free PBS (~50 mL) and concentrated using Amicon Ultra-15 Centrifugal Filters (10 kDa) (3000 g for 1–2 h, with the final volume of ~200 µL). The encapsulation efficacy (~80%) of the mRNA-LNPs was measured by an Invitrogen Quant-iT RiboGreen RNA Assay Kit (Thermo Fisher Scientific). This was followed by measurement of the endotoxin of each mRNA using an LAL Endotoxin Assay Kit (GenScript) (with a level of <1 EU/mL). About 2 µL of each encapsulated mRNA-LNP sample was loaded into the Quartz Cuvette and the relevant thermal stability and particle size were measured using DynaPro NanoStar II Light Scattering Detector (DLS) (Wyatt Technology, Santa Barbara, CA, USA).

### 2.4. mRNA Expression by Western Blot

Each mRNA-LNP (2 μg), or LNP control, was incubated with 293T cells, which were pre-seeded in a 6-well tissue culture plate, for 72 h at 37 °C. The supernatant was collected and incubated with Ni-NTA Superflow beads (40 µL) (Qiagen) overnight at 4 °C. After washing with PBS three times, the beads containing samples were boiled for 5 min in the presence of Laemmli Sample Buffer (Bio-Rad, Hercules, CA, USA). The samples were subjected to SDS-PAGE gel (7.5%) and the gel containing the expressed protein was then transferred onto the nitrocellulose membrane. After blocking with buffer (i.e., 2% fat-free milk (Bio-Rad) in PBS containing 0.05% Tween-20 (PBST)), the membrane was incubated with SARS2-S (SARS-RBD)-immunized mouse serum described below (1:500 dilution) overnight at 4 °C. After three washes, the membrane was sequentially incubated with goat-anti-mouse IgG-horseradish peroxidase (HRP, 1:60,000 dilution) (Sigma, St. Louis, MO, USA) at room temperature for 1 h and Clarity Western ECL Substrate (Bio-Rad), followed by process for images using ChemiDoc MP Imaging System (Cat. # 12003154, Bio-Rad).

### 2.5. mRNA Expression by Flow Cytometry

The expression of the mRNA-encoding target protein was carried out by flow cytometry [30,31]. Specifically, each mRNA-LNP (2 μg) or LNP control was incubated with human cell lines, including 293T, A549, or Huh-7 cells, pre-seeded in 6-well cell culture plates (2 × 10^5^/well). The cells containing each LNP-encapsulated mRNA were then cultured for 48 h at 37 °C and centrifuged at 200 g for 5 min. The cells were collected and incubated with Invitrogen-anti-His mouse-FITC antibody (Thermo Fisher Scientific). The stained cells were treated with Cytofix and Cytoperm kit (BD Biosciences, Franklin Lakes, NJ, USA) and analyzed by a flow cytometer CytoFLEX (Beckman Coulter Life Sciences, Brea, CA, USA).

### 2.6. Multiplex Assaycat

A multiplex assay was used to detect vaccine-induced cytokines in immunized mice. Specifically, splenocytes isolated from immunized mice were incubated with cell culture medium (i.e., RPMI 1640 containing 100 U/mL penicillin, 100 µg/mL streptomycin, 10% FBS, β-mercaptoethanol at 55 µM, sodium pyruvate at 1 mM, and non-essential amino acids) in the presence of mouse IL2 (1 ng/mL). To test cytokine expression, the splenocytes (1 × 10^6^ cells/mL, 200 µL/well) were incubated with 5 μg/mL of RBD-deleted SARS-CoV-2 S (for SARS-CoV-2) or SARS-CoV RBD (for SARS-CoV) protein at 37 °C [16,33]. Four days later, the cells were restimulated with the respective proteins (5 µg/mL) described above for two or more days. After centrifugation, the cell culture supernatant was collected and proceeded for measurement of cytokines using a Bio-Plex Pro Mouse Cytokine Assay kit (Bio-Rad). The results were analyzed by the Bio-Plex 200 System (Cat. # 171000205, Bio-Rad).

### 2.7. Enzyme-Linked Immunoassay (ELISA)

Vaccine-induced serum antibodies were tested by ELISA [34,35]. Specifically, RBD-deleted SARS-CoV-2 S or SARS-CoV RBD protein (1 μg/mL) was pre-coated onto 96-well ELISA plates overnight at 4℃. The plates were incubated with blocking buffer for 1 h at 37 °C and then washed with PBST three times, followed by incubation with serially diluted mouse sera for 1 h at 37 °C. The plates were washed again as described above and incubated with anti-mouse IgG-Fab (1:5000, Sigma), anti-mouse IgG1, or anti-mouse IgG2a (1:5000, Invitrogen) antibodies, which were, respectively, conjugated with HRP, for 1 h at 37℃. The plates were then incubated with the substrate 3,3′,5,5′-Tetramethylbenzidine (TMB) (Sigma) followed by the addition of H_2_SO_4_ (1 N) to stop the reaction. The absorbance values at 450 nm were obtained using Cytation 7 Microplate Multi-Mode Reader (Cat. # CYT7UMW) and Gen5 software (Gen5 iplus, v3.04.17) (BioTek Instruments, Winooski, VT, USA).

### 2.8. Pseudovirus Preparation and Pseudovirus Neutralization Assay

A safe and convenient pseudovirus neutralization assay was carried out for the evaluation of the neutralizing activity of vaccines. The pseudoviruses were generated using the following method [12,31,33,35]. Specifically, the recombinant plasmid encoding each S protein of ancestral strains of SARS-CoV-2 and SARS-CoV (GenBank accession numbers QHR63250.2 and AY274119, respectively) was mixed with two additional plasmids, including pLenti-CMV-luciferase and PS-PAX2 (Addgene, Watertown, MA, USA), using the polyetherimide (PEI reagent) (Sigma) transfection method, and the mixture was added to 293T cells. The medium from the transfected cells was removed and fresh medium was added to each well of the cells. Then, 72 h later, the supernatant containing each pseudovirus was collected and used for the pseudovirus neutralization assay described below. The sera from immunized mice were diluted at 2–5 folds and mixed with the corresponding pseudovirus for 1 h at 37 °C. The mixture of serum-virus was added to hACE2/293T target cells (i.e., 293T expressing human ACE2, the receptor of SARS-CoV-2 and SARS-CoV) at 37 °C for 24 h and the cells supplied with a fresh medium were continuously cultured for an additional 48 h. After the addition of Luciferase Cell Culture Lysis Reagent (1X) (Promega, Madison, WI, USA), the cells were lysed at room temperature with constant shaking for about 1 h at 300 rpm. After incubation with Luciferase Assay System substrate (Promega), the lysed cells were measured by Cytation 7 Microplate Multi-Mode Reader via the Luminescence Detection Method with Luminescence filter (Gain 135 and Integration time of 1 s). The relative luciferase activity was measured and the pseudovirus-neutralizing antibody titer in the tested mouse sera was reported as NT_50_ (i.e., 50% neutralizing antibody titer).

### 2.9. Plaque Reduction Neutralization Assay

The immunized mouse sera were assessed for neutralizing antibodies against infection of a heterologous SARS-CoV strain using the plaque reduction neutralization assay [6,31]. Specifically, 2-fold serially diluted mouse sera were incubated with authentic SARS-CoV (MA15 strain) for 1 h in an incubator of 37 °C and 5% CO_2_. The virus-serum mixture was added to Vero E6 cells, which were pre-seeded in 12-well plates 24 h before the experiment, and incubated for 1 h at 37 °C. The inoculations were removed from cells and the cells covered with agarose (0.6%) (Research Products International, Mt Prospect, IL, USA) were cultured in the aforementioned incubator for three additional days. The overlays of the cells were removed after incubation and the plaques in each well were stained by crystal violet (0.1%) (Thermo Fisher Scientific). The highest serum dilution being able to reduce 50% of the virus-induced plaques was reported as the NT_50_ titer.

### 2.10. Plaque Assay for the Detection of Viral Titers

Lungs from the SARS-CoV-2 or SARS-CoV-challenged mice were detected for viral titers by plaque assay [35]. Specifically, lung tissues were homogenized in PBS and collected for supernatant after centrifugation. The supernatant was then serially diluted (at 2-folds) in DMEM cell culture medium and incubated with Vero E6 cells, a cell line permissive to SARS-CoV-2 or SARS-CoV infection, pre-seeded in 12-well cell culture plates for 1 h at 37 °C. The other procedures are the same as those described in Section 2.9. Viral titers in the lungs were calculated as the PFU/mL of lung tissues.

### 2.11. Real-Time Quantitative PCR (qRT-PCR)

The qRT-PCR was applied to measure viral replication in the lungs of SARS-CoV-2-challenged mice. Specifically, lung tissues were extracted for RNAs by TRIzol reagent (Thermo Fisher Scientific), which was utilized as the template for amplification of cDNA. The qRT-PCR was carried out by Master Mix reagent (Power SYBR Green PCR Master Mix) (Applied Biosystems, Waltham, MA, USA). The amplified nucleocapsid (N) gene was normalized to GAPDH (i.e., glyceraldehyde-3-phosphate dehydrogenase) (ΔCT = threshold cycle (CT) of the N gene − CT of GAPDH). The results are presented as the ratio to GAPDH (calculated as 2^−ΔCT^).

### 2.12. Mouse Immunization Procedures

BALB/c (aged 6–8 weeks) were subjected to immunization and challenge. Groups of five mice were assigned to each group in a random fashion and immunized with the well-characterized mRNA vaccines or control as described below using the intradermal (i.d.) route, an injection route previously optimized for mRNA vaccines [12,31]. Specifically, mice were i.d. vaccinated with SARS2-S (SARS-RBD) mRNA-LNPs, SARS2-S (RBD-del) mRNA-LNPs (10 μg/mouse in 100 μL), or an LNP control (100 μL/mouse). Each mouse was boosted twice at 3-week intervals as described above. The mice were bled 10 days after the last immunization and the sera were tested by ELISA for measurement of IgG antibodies or IgG1 and IgG2a subtype antibodies, specific to the S or RBD protein, as well as by neutralization assays for calculation of neutralizing antibodies. Splenocytes collected 2 months post-last immunization were evaluated for T-cell cytokine expression by Multiplex assay as described above.

### 2.13. Challenge of Immunized Mice with SARS-CoV-2 Omicron Variant

The challenge of immunized mice with SARS-CoV-2 was performed as described below [6,12,34]. Specifically, 40 days post-last immunization, anesthetized mice were intranasally (i.n.) challenged with an optimal dose of SARS-CoV-2 Omicron (BA.1) variant (B.1.1.529: 10^5^ plaque-forming unit (PFU)/mouse in 50 μL). The mice were euthanized two days post-challenge, and the lung tissues were harvested for detection of viral titers by plaque assay and viral replication by qRT-PCR as described above.

### 2.14. Challenge of Immunized Mice with SARS-CoV

The challenge with SARS-CoV was carried out as described below. Specifically, 40 days after the last immunization, mice were anesthetized, intranasally (i.n., an optimal route for SARS-CoV) infected with SARS-CoV (mouse-adapted MA15 strain, 500 PFU/mouse in 50 μL volume: a lethal infectious dose previously identified for BALB/c mice), and then observed for survival and body weight loss for 14 days after infection.

### 2.15. Challenge of Immune Serum-Transferred Mice with SARS-CoV

The passive protective immunity was tested as below. Naïve mice were intraperitoneally (i.p.) injected with pooled sera of mice collected 10 days after the last immunization of each mRNA vaccine or LNP control (200 μL/mouse). Then, 12 h later, these mice were intranasally (i.n.) infected with SARS-CoV (mouse-adapted MA15 strain, 400 PFU/mouse in 50 μL volume: an optimal dose for measurement of passive protective efficacy and viral titers in the preliminary studies). Two days after infection, lungs were harvested from each mouse and detected for viral titers by plaque assay as described above.

### 2.16. Statistical Analysis

GraphPad Prism 9 statistical software was applied in this study to analyze statistical significance among different groups. Ordinary one-way ANOVA (Tukey’s multiple comparison test) was applied to calculate the statistical significance of T-cell responses, lung viral titers, and viral replication among different groups. An unpaired two-tailed student *t*-test was applied to calculate the statistical significance of weight changes in mice between two groups. *p* < 0.05, *p* < 0.01, and *p* < 0.001 are expressed as *, **, and ***, respectively.

## 3. Results

### 3.1. Construction and Characterization of mRNA Vaccines

**Figure 1:**
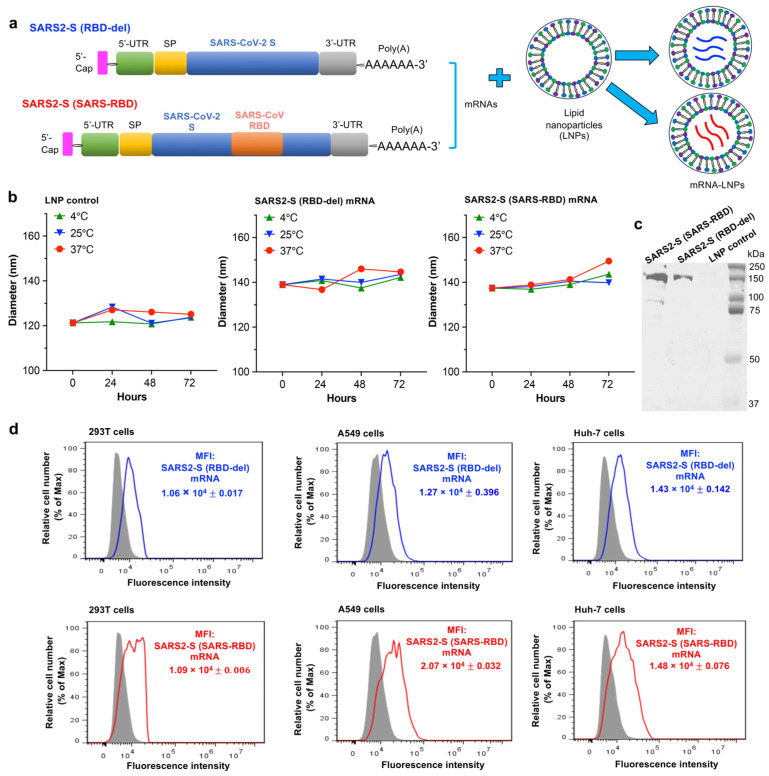
Construction and characterization of mRNA vaccines. (**a**) Construction of nucleoside-modified SARS2-S (SARS-RBD) and SARS2-S (RBD-del) mRNAs. Each of the synthesized mRNAs encodes tissue plasminogen activator (tPA) signal peptide (SP) and SARS-CoV-2 Omicron spike (S) protein with a deleted receptor-binding domain (RBD) or with the inserted SARS-CoV RBD, and contains a 5′ cap, 5′ untranslated region (UTR), 3′-UTR, and 3′ poly(A) tail. The synthesized mRNAs were formulated with lipid nanoparticles (LNPs) for vaccine delivery. (**b**) Detection of the stability of LNP-formulated mRNA vaccines or LNP control under different conditions. mRNA vaccines or control were stored at 4 °C, 25 °C, and 37 °C for 0, 24, 48, and 72 h, respectively, followed by measurement of particle sizes (hydrodynamic diameter) by DLS. (**c**) Western blot for detection of expression of mRNA-encoding protein. Each mRNA-LNP was incubated with 293T cells for 72 h at 37 °C and the culture supernature was tested by SARS2-S (SARS-RBD)-immunized mouse sera. kDa, protein molecular weight marker. (**d**) Representative figures of flow cytometry analysis of expression of mRNA-encoding protein. Each mRNA-LNP was incubated with 293T, A549, and Huh-7 cells, respectively, for 48 h at 37 °C, which were then stained with FITC-labeled mouse-anti-His antibody and measured for fluorescence intensity by flow cytometry. The grey-shaded region represents LNP-incubated cell controls. MFI indicates the Median Fluorescence Intensity. The data are expressed as mean ± standard deviation of the mean (s.e.m.) of triplicate wells. One experimental repeat was performed and similar results were obtained.

Two mRNA vaccines were constructed for this study. SARS2-S (RBD-del) mRNA was constructed to encode the S protein (without RBD) of the SARS-CoV-2 Omicron variant with HexaPro sequences (Figure 1a). SARS2-S (SARS-RBD) mRNA was constructed by inserting the RBD of SARS-CoV into the above construct (Figure 1a). Each mRNA cassette on a pCAGGS vector also contains an N-terminal tPA signal peptide, a C-terminal foldon trimeric sequence, and a C-terminal His_6_ tag. The constructed mRNAs were synthesized using the MEGAscript T7 Transcription Kit in the presence of nucleosides (ATP, GTP, and CTP) and a modified nucleoside (Pseudo-UTP) to increase stability and remove mRNA-associated innate immune activation, as shown in our previous studies [12,30]. The synthesized mRNAs also contain an N-terminal 5′-untranslated region (5′-UTR) and a 3′-UTR, as well as an N-terminal Cap sequence and a C-terminal poly(A) tail (Figure 1a). To further improve their stability, the synthesized mRNAs were formulated with LNPs and the mRNA-LNPs were used as vaccines (Figure 1a). The sizes of the LNP-formulated SARS2-S (SARS-RBD) and SARS2-S (RBD-del) mRNAs, which were measured by the DLS, ranged from around 140–150 nm, with these sizes remaining similar at 4 °C and after treatment at 25 °C and 37 °C, respectively, for 24, 48, and 72 h (Figure 1b). Western blot analysis identified a clear band in the mRNA-LNP-incubated 293T cell culture supernatant, not in the cell culture supernatant incubated with the LNP control, confirming the expression of S-specific protein (Figure 1c). Flow cytometry analysis indicated that these mRNAs expressed His-tagged proteins efficiently in human cell lines, including 293T, A549, and Huh-7 cells (Figure 1d). These data indicated that LNP-formulated mRNA vaccines were stable and able to efficiently express target proteins in different human cells.

### 3.2. SARS2-S (SARS-RBD) mRNA Vaccine Elicited Effective Antibodies with Neutralizing Activity against SARS-CoV-2 and SARS-CoV

**Figure 2:**
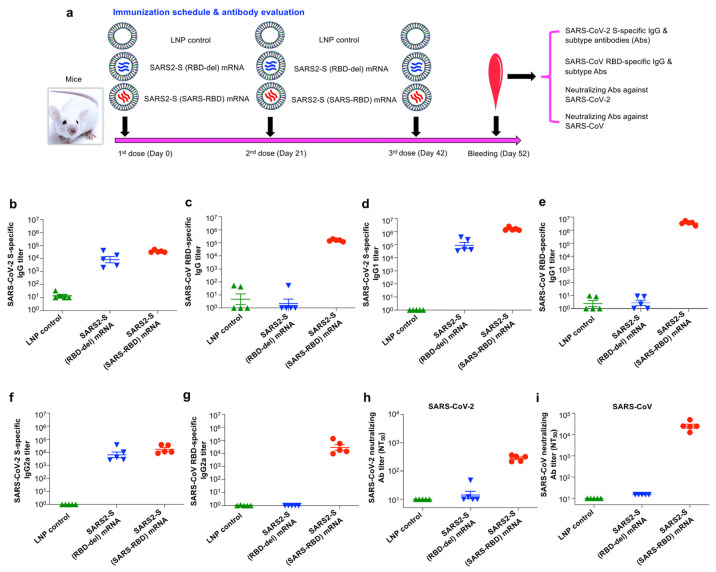
Evaluation of antibody responses and neutralizing antibodies induced by mRNA vaccines against infection of SARS-CoV-2 and SARS-CoV. (**a**) BALB/c mice were vaccinated with each mRNA vaccine, or LNP control, for a total of 3 times at 3-week intervals and bled 10 days post-last vaccination to test, by ELISA, for serum IgG antibodies targeting the RBD-deleted SARS-CoV-2 S (**b**) or SARS-CoV RBD (**c**) protein. These sera were also evaluated, by ELISA, for serum IgG1 subtype antibodies targeting the aforementioned SARS-CoV-2 S (**d**) or SARS-CoV RBD (**e**) protein, as well as for serum IgG2a subtype antibodies targeting the aforementioned SARS-CoV-2 S (**f**) or SARS-CoV RBD (**g**) protein. The SARS-CoV-2 S or SARS-CoV RBD protein was pre-coated to the ELISA plates and the respective antibody (Ab) titer was reported as mean ± s.e.m. (from five mice per group). The aforementioned mouse sera were also detected for neutralizing antibodies against pseudoviruses expressing the respective S proteins of SARS-CoV-2 ancestral strain (**h**) and the ancestral strain (Tor2) of SARS-CoV (**i**). The neutralizing antibody titer (NT_50_: 50% neutralizing Ab titer) is expressed as mean ± s.e.m. (from five mice per group). One experimental repeat was performed and similar results were obtained.

To investigate the immunogenicity of these mRNA vaccines, as shown by their ability to induce specific antibody responses, BALB/c mice were vaccinated with each mRNA (encapsulated with LNPs) or LNPs alone as a control. Sera were taken from each mouse after the last immunization and measured for specific IgG antibodies, including subtype (IgG1 and IgG2a) by ELISA and neutralizing antibody titers by a pseudovirus neutralization assay (Figure 2a). SARS2-S (SARS-RBD) mRNA induced similar levels of IgG antibodies specific to both SARS-CoV-2 S and SARS-CoV RBD proteins, whereas SARS2-S (RBD-del) mRNA only induced IgG antibodies that reacted with SARS-CoV-2 S protein, not SARS-CoV RBD protein (Figure 2b,c). In addition, SARS2-S (SARS-RBD) mRNA induced potent IgG1 and IgG2a subtype antibodies that were reactive to both the SARS-CoV-2 S and SARS-CoV RBD proteins, whereas SARS2-S (RBD-del) mRNA induced IgG1 and IgG2a antibodies that targeted only the S protein of SARS-CoV-2, not the RBD protein of SARS-CoV (Figure 2d–g). Moreover, SARS2-S (SARS-RBD) mRNA induced potent and high-titer neutralizing antibodies, being able to potently neutralize infection with pseudotyped SARS-CoV encoding the S protein of an ancestral Tor2 strain, and effective neutralizing antibodies, which were able to neutralize infection of pseudotyped SARS-CoV-2 encoding the S protein of the ancestral strain (Figure 2h,i). By comparison, SARS2-S (RBD-del) mRNA induced low-level neutralizing antibodies against infection of these pseudoviruses tested (Figure 2h,i). LNP alone (control) failed to induce specific antibodies that neutralized SARS-CoV-2 and SARS-CoV pseudovirus infections (Figure 2). These results indicated that SARS2-S (SARS-RBD) mRNA was able to elicit strong antibodies with neutralizing activity against infection by SARS-CoV-2 and SARS-CoV ancestral strains.

### 3.3. SARS2-S (SARS-RBD) mRNA Vaccine Elicited Durable SARS-CoV-2 S and SARS-CoV RBD-Specific T-Cell Responses

**Figure 3:**
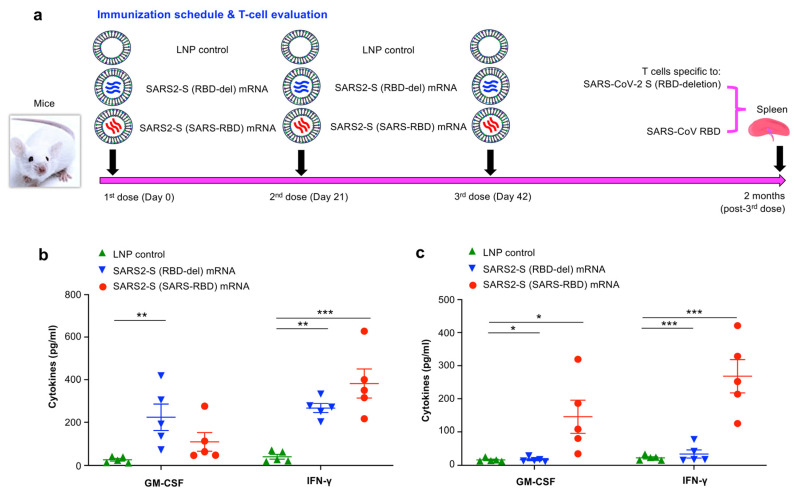
Evaluation of mRNA vaccine-induced T-cell responses. (**a**) BALB/c mice were immunized with each mRNA vaccine or LNP control for 3 times at 3-week intervals and splenocytes collected 2 months post-last vaccine dose were tested for cytokine expression by Multiplex assay. Isolated splenocytes were stimulated with RBD-deleted SARS-CoV-2 S (**b**) or SARS-CoV RBD (**c**) protein and the expressed cytokines (pg/mL) were measured in the supernatant. Significant differences among different groups are shown as * (*p* < 0.05), ** (*p* < 0.01), and *** (*p* < 0.001). The experiments were repeated once, resulting in similar results.

To investigate the ability of these mRNA vaccines to elicit durable and specific T-cell responses, splenocytes were isolated from vaccinated BALB/c mice 2 months post-last immunization and stimulated with the RBD-deleted SARS-CoV-2 S or SARS-CoV RBD protein and cytokine expression was measured by Multiplex assay (Figure 3a). Both SARS2-S (SARS-RBD) and SARS2-S (RBD-del) mRNAs induced SARS-CoV-2 S-specific IFN-γ response, with these responses being significantly higher than those induced by the LNP control (Figure 3b). In addition, SARS2-S (RBD-del) mRNA induced a significantly higher level of GM-CSF than that induced by the LNP control (Figure 3b). Unlike SARS2-S (RBD-del) mRNA, SARS2-S (SARS-RBD) induced significantly higher levels of GM-CSF and IFN-γ cytokines specific to SARS-CoV RBD (Figure 3c). These findings suggested that SARS2-S (SARS-RBD) mRNA was able to induce long-term SARS-CoV-2 S and SARS-CoV RBD-specific T-cell responses, whereas SARS2-S (RBD-del) mRNA induced only anti-SARS-CoV S, but not anti-SARS-CoV RBD, T-cell responses.

### 3.4. SARS2-S (SARS-RBD) mRNA Vaccine-Protected Mice against Challenge with SARS-CoV-2 Omicron Variant and SARS-CoV

**Figure 4:**
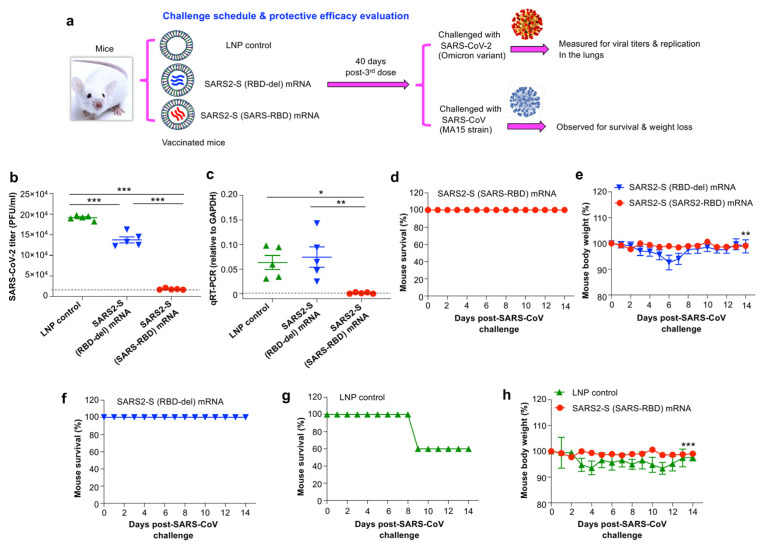
Evaluation of mRNA vaccine-induced protective efficacy against SARS-CoV-2 and SARS-CoV. (**a**) At a time of 40 days after the last immunization, BALB/c mice were i.n.-challenged with the SARS-CoV-2 Omicron variant (BA.1, 10^5^ PFU/mL) and lungs were collected two days later to measure viral titers by plaque assay and viral replication by qRT-PCR. Evaluation of viral titers (**b**) and viral replication (**c**) in the lungs. The viral titers were reported as the PFU/mL of lung tissues. The viral (nucleocapsid gene) replication was normalized to glyceraldehyde-3-phosphate dehydrogenase (GAPDH). The data are presented as mean ± s.e.m. (from five mice per group). One experimental repeat was performed and similar results were obtained. (**d**–**h**) In a separate experiment, vaccinated mice were challenged (i.n.) with the MA15 strain of SARS-CoV (500 PFU/mL) and investigated for survival and weight loss for 14 days after the virus challenge. The data (in (**e**) and (**h**)) are expressed as mean ± s.e.m. of three (for surviving mice in the LNP control group from day 9) to five mice (for mRNA vaccine groups and LNP control group by day 8) in each group. Significant differences among different groups are shown as * (*p* < 0.05), ** (*p* < 0.01), and *** (*p* < 0.001).

To evaluate the protective efficacy of these mRNA vaccines, BALB/c mice immunized with each mRNA or LNP control were separately challenged with the Omicron (BA.1) variant of SARS-CoV-2 or the mouse-adapted MA15 strain of SARS-CoV 40 days after the last dose of vaccine (Figure 4a). Since the Omicron variant of SARS-CoV-2 was not lethal to wild-type mice, protective efficacy was evaluated by measuring viral titers by plaque assay and viral replication by qRT-PCR assay after the virus challenge. Instead, survival and weight changes were determined in the mice challenged with SARS-CoV. SARS2-S (SARS-RBD) mRNA potently inhibited SARS-CoV-2 infection, with viral titers and viral replication being significantly lower in the lungs of challenged mice than in the lungs of mice immunized with SARS2-S (RBD-del) mRNA or LNP control (Figure 4b,c). In addition, SARS2-S (RBD-del) mRNA inhibited SARS-CoV-2 infection to some extent, with viral titers being significantly lower in the lungs of immunized mice than in mice receiving LNP control (Figure 4b). SARS2-S (SARS-RBD) mRNA also completely protected immunized mice against the SARS-CoV challenge, with a survival rate of 100% and an absence of weight loss over 14 days (Figure 4d,e). Although SARS2-S (RBD-del) mRNA fully protected immunized mice against death from the SARS-CoV challenge, significant weight loss was observed in these mice, particularly 5–7 days after infection (Figure 4e,f). By contrast, 40% of mice injected with LNP control did not survive the SARS-CoV challenge, leading to significant weight loss, particularly 3–11 days after infection (Figure 4g,h). These findings indicated that SARS2-S (SARS-RBD) mRNA was able to fully prevent infection with both SARS-CoV-2 and SARS-CoV.

### 3.5. SARS2-S (SARS-RBD) mRNA-Immune Sera Protected Naïve Mice against SARS-CoV-2 Infection

**Figure 5:**
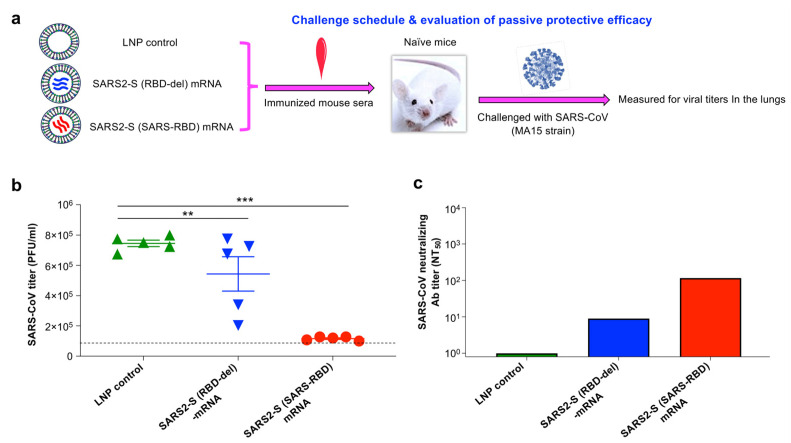
Evaluation of passive protective efficacy of mRNA vaccine-induced mouse serum antibodies. (**a**) Experimental procedure and challenge schedule. Naïve BALB/c mice were i.p.-injected with the pooled sera of mice receiving vaccines (SARS2-S (SARS-RBD) mRNA or SARS2-S (RBD-del) mRNA) or LNP control, i.n.-challenged with the heterologous SARS-CoV (MA15 strain, 400 PFU/mL) 12 h later, and measured for viral titers in the lungs by plaque assay two days post-challenge. (**b**) Evaluation of viral titers after serum transfer. The viral titers were expressed as the PFU/mL of lung tissues. The data are presented as mean ± s.e.m. (from five mice per group). ** (*p* < 0.01) and *** (*p* < 0.001) indicate significant differences among different groups. (**c**) Plaque reduction neutralization assay was tested for the aforementioned pooled mouse sera against infection of the heterologous authentic MA15 strain of SARS-CoV. The 50% neutralizing antibody (Ab) titer (NT_50_) was calculated and the data are expressed as mean ± s.e.m. (from duplicate wells of pooled sera per group). One experimental repeat was performed and similar results were obtained.

The ability of SARS2-S (SARS-RBD) mRNA to strongly induce serum-neutralizing antibodies against SARS-CoV infection in vitro suggested that these serum antibodies may be effective against SARS-CoV infection in vivo. To test this and also to evaluate the broad-spectrum efficacy of the designed mRNA vaccine against another SARS-CoV strain, pooled serum from mice immunized with each mRNA or LNP control was passively transferred to naïve BALB/c mice, which were challenged with the MA15 strain of SARS-CoV. Two days later, viral titers were measured by plaque assay in the lungs of these mice (Figure 5a). Viral titers were significantly lower in the recipient lungs of mice that were passively transferred with sera from mice immunized with SARS2-S (SARS-RBD) mRNA than in lungs of mice receiving transferred sera from mice immunized with SARS2-S (RBD-del) mRNA and mice injected with LNP alone (Figure 5b). In addition, the sera of mice immunized with SARS2-S (SARS-RBD) mRNA were found to contain antibodies with effective neutralizing activity against infection of this authentic heterologous SARS-CoV (MA15) (Figure 5c). By contrast, the low-titer neutralizing antibodies in the sera of mice immunized with SARS2-S (RBD-del) mRNA were not sufficient to protect against the SARS-CoV challenge, with three of five mice that were passively transferred with these sera having high-level lung viral titers similar to findings in mice receiving passively transferred sera from LNP control mice (Figure 5b-c). These results indicated that serum antibodies of mice immunized with SARS2-S (SARS-RBD) mRNA significantly prevented infection of the heterologous SARS-CoV, MA15 strain, in naïve mouse recipients, with the level of protection being positively correlated with neutralizing antibody titers in their sera.

## 4. Discussion

SARS-CoV-2, particularly the newly emerging variants, continues to infect humans and continues to show broad and rapid transmissibility [9,36]. The generation of new mutations in the amino acid sequences of the RBD and other important vaccine targets of SARS-CoV-2 have caused, and may continue to result in the future emergence of, additional variant(s) that are significantly, or completely, resistant to the immune protection provided by current vaccines or therapeutic antibodies targeting these regions [21,37,38,39,40,41]. In addition, SARS-CoV and SARS-like CoVs from other species use the same cellular receptor as SARS-CoV-2 for viral entry [1,42,43,44], thereby having pandemic potential. Safe and effective vaccines are therefore needed to prevent infection with SARS-CoV-2 and related CoVs with pandemic potential.

The emergence of recurrent SARS-CoV-2 variants with enhanced ability to escape immune responses and/or protection provided by previous vaccines will require the development of new vaccines adapted to these new variants or the generation of bivalent vaccines targeting the ancestral strain and new variants. For example, bivalent vaccines encoding the S proteins of wild-type SARS-CoV-2 strain and the Beta, Delta, or Omicron variants were approved or authorized for clinical use [45,46,47,48]. However, the development of vaccines targeting current or future new variants is both time-consuming and costly. A universal vaccine that can protect against multiple SARS-CoV-2 variants and other CoVs would preclude the need to develop vaccines against newly emergent CoV strains and may have the potential to prevent future pandemics. Development and stockpiling of a pan-coronavirus vaccine would be beneficial significantly to both public health and the global economy [49].

mRNA vaccines possess a variety of advantages, from production to manufacturing, leading to fast approval of at least two of such vaccines for preventing SARS-CoV-2 infection and stopping the COVID-19 pandemic [50,51]. Currently, mRNA-based COVID-19 vaccines are developed targeting SARS-CoV-2 S protein or its fragment RBD, as well as other viral proteins such as N [26,27,52,53,54]. While most of these mRNA vaccines induced systemic immune responses and/or protection against SARS-CoV-2 infection via the intramuscular route, other vaccines may induce mucosal immunity via different injection routes such as the intranasal route to deliver the mRNA to the nasal mucosa or lungs [55,56]. To improve the breadth of neutralization and potency of the vaccines against heterologous or multiple variant infections, bivalent vaccines (by mixing two mRNAs encoding S protein of ancestral strain or different variants) or priming-boosting vaccination strategies (by priming with a mRNA targeting ancestral or a variant and boosting with monovalent or bivalent mRNA, or a viral vector, targeting ancestral or one or two variants) are employed [45,47,57,58].

The present study designed a unique mRNA vaccine, SARS2-S (SARS-RBD), which protected against both SARS-CoV-2 and SARS-CoV infections. This mRNA vaccine was designed to target the conserved region of the SARS-CoV-2 S protein (without its own RBD) and encode the critical neutralizing region of SARS-CoV RBD [16]. SARS2-S (SARS-RBD) efficiently expressed the related protein in several human cell lines. It was stable under several temperatures tested (i.e., 4 °C, 25 °C, and 37 °C) in the detection period of up to 72 h, partially due to the use of the modified nucleoside (Pseudo-UTP) during the mRNA synthesis and further encapsulation of the synthesized mRNA with the LNPs. Future studies will be performed to investigate the stability of the mRNA at high temperatures (>37 °C) and over a long period (for up to several months to one year). This mRNA vaccine did not show obvious side effects during vaccination, and i.d. delivery of the vaccine elicited potent antibodies and T-cell responses specific to both SARS-CoV-2 S and SARS-CoV RBD proteins. Notably, the protective efficacy of the designed mRNA vaccine was confirmed in mice with promising results. This vaccine-induced broadly protective efficacy is capable of significantly protecting immunized mice against challenges with the SARS-CoV-2 Omicron variant and SARS-CoV. Importantly, passive transfer of serum antibodies from immunized mice protected naïve mouse receipts against SARS-CoV-infection, with the level of protection correlating positively with a serum-neutralizing antibody titer. There was about 70% amino acid similarity between the RBD sequences of SARS-CoV and SARS-CoV-2 (Omicron variant). Different from the SARS2-S (SARS-RBD) mRNA described above, which contains the RBD of SARS-CoV, SARS2-S (RBD-del) mRNA, which lacks the SARS-CoV RBD, resulted in the elicitation of antibodies and T-cell responses specific to SARS-CoV-2 S, rather than to SARS-CoV RBD, partially protecting immunized mice from the SARS-CoV-2 Omicron variant and SARS-CoV infection.

Of note, SARS2-S (SARS-RBD) mRNA elicited broad-spectrum neutralizing activity against infection of the pseudotyped SARS-CoV-2 ancestral strain, as well as two strains of SARS-CoV tested (i.e., pseudotyped ancestral and authentic mouse-adapted strains). CoV pseudoviruses, which do not contain their viral infectious components with single-cycle infection, can be generated by co-transfection of a plasmid encoding viral surface S protein and one or two accessory plasmids for providing a report gene or other necessary components [30]. Different from the live virus neutralization assay, the pseudovirus neutralization assay is safe and convenient for evaluating the neutralizing activity of the designed vaccines without the requirement of BSL-3 facilities. The neutralizing antibody titer from the pseudotyped CoV neutralization assay is generally higher than that from the live virus neutralization assay but the results are overall consistent in both neutralization assays in terms of the same viral strain tested [12].

The mouse model is the most common model for evaluating the protective efficacy of COVID-19 and SARS vaccines due to its convenience, cost-effectiveness, and easy and rapid manipulation of viral infection. The vaccine-induced protective efficacy in mice is generally determined by increased survival, and reduced clinical signs, viral loads, or pathological changes in lungs after virus challenge, depending on the virus strains and mouse models used [59,60,61]. Notably, vaccines with effective neutralizing activity and/or protective efficacy in mice are most likely to present effective neutralizing immunity in humans [59,62,63,64]. The hamster model is another animal model widely used for SARS-CoV-2 challenge studies. SARS-CoV-2 is not lethal to hamsters, so the protection is normally evaluated for reduced clinical symptoms, decreased viral titers (in the respiratory tract, nasal turbinate, and lungs) and lung pathology, or prevention of virus transmission [56,65,66,67]. This study used wild-type mice to evaluate the protective efficacy of the designed mRNA vaccine since these mice are susceptible to SARS-CoV-2 Omicron variants and the SARS-CoV MA15 strain tested. The candidate vaccine will be investigated for its efficacy in other animal models such as hamsters in future studies to compare the correlation of the neutralizing activity and protective efficacy in different challenge models.

Overall, the LNP-encapsulated SARS2-S (SARS-RBD) mRNA vaccine described in this study demonstrated broad-spectrum efficiency to potently prevent infection with the SARS-CoV-2 Omicron (BA.1) variant and the SARS-CoV strain, without the need to alter or modify its sequences. The current study did not evaluate the efficiency of the designed mRNA vaccine against other SARS-CoV-2 variants or SARS-related CoVs. Due to the high sequence conservation of the non-RBD S region among different SARS-CoV-2 strains, the designed mRNA, which is based on this conserved S protein region, is expected to protect against other SARS-CoV-2 strains. Further studies will be conducted to evaluate the protective efficacy of this vaccine against additional SARS-CoV-2 strains, including the current dominant Omicron JN.1 or KP.2 variant, as well as its cross-protective efficacy against other CoVs in the same beta-CoV genus as SARS-CoV-2 and SARS-CoV.

## 5. Conclusions

The present study described a unique mRNA vaccine with cross-neutralizing activity against the SARS-CoV-2 original strain and protective efficacy to prevent infection of an earlier SARS-CoV-2 Omicron variant, as well as SARS-CoV. This mRNA vaccine will be tested for its potential to protect against current dominant or future SARS-CoV-2 variants and perhaps other SARS-related CoVs with pandemic potential.

## 6. Patents

The authors have filed a patent application related to this study with L.D. and G.W. as inventors.

## Data Availability

All data related to this study are included in this article. No special code was used in the study. Special constructs and materials will be made available under a Material Transfer Agreement (MTA).
